# Suitability of magnetic nanoparticle immobilised cellulases in enhancing enzymatic saccharification of pretreated hemp biomass

**DOI:** 10.1186/1754-6834-7-90

**Published:** 2014-06-11

**Authors:** Reinu E Abraham, Madan L Verma, Colin J Barrow, Munish Puri

**Affiliations:** 1 Centre for Chemistry and Biotechnology (CCB), Geelong Technology Precinct, Waurn Ponds, Deakin University, Geelong, Victoria 3217, Australia

**Keywords:** Hemp hurd, Cellulase, Immobilisation, Nanoparticle, Enzyme, Hydrolysis

## Abstract

**Background:**

Previous research focused on pretreatment of biomass, production of fermentable sugars and their consumption to produce ethanol. The main goal of the work was to economise the production process cost of fermentable sugars. Therefore, the objective of the present work was to investigate enzyme hydrolysis of microcrystalline cellulose and hemp hurds (natural cellulosic substrate) using free and immobilised enzymes. Cellulase from *Trichoderma reesei* was immobilised on an activated magnetic support by covalent binding and its activity was compared with that of the free enzyme to hydrolyse microcrystalline cellulose and hemp hurds on the basis of thermostability and reusability.

**Results:**

Up to 94% protein binding was achieved during immobilisation of cellulase on nanoparticles. Successful binding was confirmed using Fourier transform infrared spectroscopy (FTIR). The free and immobilised enzymes exhibited identical pH optima (pH 4.0) and differing temperature optima at 50°C and 60°C, respectively. The K_*M*_ values obtained for the free and immobilised enzymes were 0.87 mg/mL and 2.6 mg/mL respectively. The immobilised enzyme retained 50% enzyme activity up to five cycles, with thermostability at 80°C superior to that of the free enzyme. Optimum hydrolysis of carboxymethyl cellulose (CMC) with free and immobilised enzymes was 88% and 81%, respectively. With pretreated hemp hurd biomass (HHB), the free and immobilised enzymes resulted in maximum hydrolysis in 48 h of 89% and 93%, respectively.

**Conclusion:**

The current work demonstrated the advantages delivered by immobilised enzymes by minimising the consumption of cellulase during substrate hydrolysis and making the production process of fermentable sugars economical and feasible. The activity of cellulase improved as a result of the immobilisation, which provided a better stability at higher temperatures. The immobilised enzyme provided an advantage over the free enzyme through the reusability and longer storage stability properties that were gained as a result of the immobilisation.

## Introduction

The increasing global dependence on fossil fuels, combined with their increasing cost and gradual depletion, is driving the search for alternatives to fossil-based energy sources. This search has resulted in growing interest in the production of ethanol from lignocellulosic biomass, a natural and renewable agricultural and industrial waste product [1] whose cellulosic polymers can be converted into fermentable sugars to produce ethanol [2]. Lignocellulose is a complex carbohydrate polymer interconnected with strong bonds that give it a highly robust structure. Cellulases are a group of complex enzymes that catalyse the hydrolysis of cellulose and exhibit synergistic action [3]. Due to the structural complexity of this biomass, the synergistic actions of the endoglucanase, exoglucanase and beta-glucosidase enzymes of the cellulase group are required for hydrolysis [4-6]. These three enzymes break the cross-linked bonds in cellulose and produce monomers of glucose for fermentation.

Biomass is available in the form of hardwoods and softwoods, and agricultural and industrial waste [7]. The use of these renewable, readily available and noncompeting fuel sources for the production of energy presents a solution both to depleting energy reserves and the treatment of existing waste. A large amount of cellulosic waste is produced from the textile and fibre industries. This waste can be further utilised for bioenergy production, thus adding value to the material. Hemp (*Cannabis sativa*) hurd biomass (HHB) is easily available due to its extensive application in the fibre and textile industry. Hemp is an annual herbaceous crop which exhibits both bast fibre and a woody core [8], the former of which finds a host of applications in industry. The remaining woody core is typically considered a waste product, making it an ideal candidate source of cheap, readily available cellulose for the production of fermentable sugars to produce ethanol [9]. The pretreatment of biomass, which includes the removal of lignin and the opening of the structure, is a key step in the bioconversion process of biomass to ethanol, as it enables efficient enzyme access and biomass hydrolysis, resulting in high yields of reducing sugars [10]. Various potentially bottlenecking steps, such as breaking the complex lignocellulose structure, enzyme loading, interference of inhibitors during hydrolysis, and fermentation, are all necessary components of the bioconversion process, although the past few years of research have resulted in an array of solutions to mitigate their negative impacts on process efficiency [11].

Immobilisation enhances the biocatalytic properties of an enzyme, including stability and reusability [12]. The binding of enzymes onto a nanosized magnetic particle provides better separation from the reaction mixture. Previous reports have suggested that these magnetic supports are less toxic and provide a higher surface area, and they are now finding application in medical, textile and waste recycling processes [13]. In bioenergy production, the immobilisation of enzymes onto nanomaterials has the potential to improve the economic viability of the entire process [14]. There are various advantages of immobilised enzymes over free enzymes, including thermostability, enzyme reusability and storage, making them suitable as superior free enzyme substitutes for a host of applications. The activated nanosized support provides surface area and strong cross-linking through covalent bonds [15]. Enzymes such as beta-glucosidase, which can be used for the hydrolysis of lignocellulosic biomass, have been immobilised on various supports to successfully improve their biochemical properties and stability. Studies have also been conducted in recent years to immobilise cellobiase for hydrolysing pretreated biomass [16,17].

The present work focuses on the hydrolysis of the microcrystalline carboxymethyl cellulose (CMC) as well as natural cellulosic biomass (HHB) using an immobilised enzyme. Immobilisation of cellulase onto an activated magnetic nanoparticle was achieved using glutaraldehyde as a cross-linker. Being magnetic in nature, a magnetic nanoparticle provides the advantage of easy separation of the immobilised enzyme from the reaction mixture. After thorough washing this separated immobilised mixture can be reused. The biochemical characterisation of the free and immobilised enzyme at different temperatures and substrate concentrations was investigated. The catalytic efficiency of immobilised cellulase was assessed based on its thermostability, reusability and storage.

## Materials and methods

### Materials

#### Chemicals

The present study utilised recombinant cellulase (EC 3.2.1.4; 700 units) from *Trichoderma reesei,* ferric chloride, zinc chloride, potassium hydrogen phthalate, sodium acetate, sodium citrate, potassium phosphate, Trizma hydrochloride and CMC procured from Sigma-Aldrich. Glutaraldehyde was procured from SAFC Supply Solutions. The protein assay kit (Bio-Rad protein dye reagent concentrate) was sourced from Bio-Rad.

#### Cellulosic biomass

The biomass used for the study was hemp hurd (*Cannabis sativa*), procured as an industrial residue. The biomass was milled using a Fritsch Pulverisette 19 Universal Cutting Mill. The milled biomass was sieved using a mesh of pore size about 300 μm.

### Nanomaterial

The strong magnetic properties of nanoparticles assist in the efficient recovery of the immobilised enzyme. To increase the saturation magnetisation of nanoparticles, zinc was doped into magnetite for the present study. Magnetic nanoparticles were synthesised using a hydrothermal method. To achieve this, aqueous solutions of iron(III) chloride hexahydrate (FeCl_3_.6H_2_O), iron(II) chloride tetrahydrate (FeCl_2_.4H_2_O) and zinc chloride (ZnCl_2_) were mixed in a molar ratio of Fe^3+^:Fe^2+^:Zn^2+^ = 2.0:0.6:0.4. An aqueous sodium hydroxide (NaOH) solution was subsequently added to neutralise the pH. The precipitates were subjected to hydrothermal treatment at 150°C for 12 h, followed by repeated rinsing with deionised water and freeze-drying at -80°C and 0.014 mbar for 24 h.

The crystalline structure of the nanopowder was characterised using an X’Pert pro X-ray diffractometer (Pan-Analytical, The Netherlands) with Cu K-alpha radiation (40 KV, 30 mA). The morphology of the synthesised particles was characterised by transmission electron microscopy (TEM) using a JEOL 2100 M microscope (JEOL, Japan) with an electron beam energy of 200 kV. The magnetic hysteresis of the particles was measured using a semiconductor quantum interference device magnetometer (Quantum Design Inc., San Diego, CA, USA) at room temperature.

#### Immobilisation of cellulase on the activated magnetic nanoparticle

The magnetic nanoparticles were suspended in deionised water at a concentration of 5 mg/mL. This suspension was sonicated for 1 h, after which it was suspended in 1 M glutaraldehyde solution in deionised water [18]. Support activation was achieved by incubating the magnetic nanoparticles for 1 h at 25°C in a shaker at 250 rpm. The activated magnetic support was washed twice with deionised water and once with sodium acetate buffer.

The covalent binding of the enzyme to the nanoparticles was achieved by incubating the activated nanoparticle support with enzyme at a concentration of 5 mg/mL at 25°C for 2 h in a shaker at 250 rpm. The supernatant obtained after separating the immobilised mixture from solution was used for protein estimation. The immobilised enzyme on the nanoparticle support was thoroughly washed with deionised water and buffer to remove any loosely bound protein.

The binding efficiency of the enzyme was determined by calculating the ratio of total protein bound, as determined by the Bradford assay, to the total protein available for immobilisation:

$$\mathrm{Binding}\;\mathrm{efficiency}\left(\%\right)=\frac{\mathrm{Total}\;\mathrm{amount}\;\mathrm{of}\;\mathrm{protein}\;\mathrm{binded}}{\mathrm{Total}\;\mathrm{amount}\;\mathrm{of}\;\mathrm{protein}\;\mathrm{added}}*100$$

#### Enzyme assay

The enzyme assay for free and immobilised enzymes was carried out using a CMC assay [19]. The assay for the free enzyme was conducted at 50°C with a reaction mixture containing 0.5 mL enzyme (about 20 CMC units) and 0.5 mL of 2% substrate (CMC) dissolved in 0.1 M sodium acetate buffer (pH 4.0) and incubated for 30 min. The reaction was stopped by adding 3 mL of DNS reagent and heating for 10 min in a vigorously boiling water bath. The concentrations of glucose released were measured at 540 nm. The estimation of reducing sugars produced during enzyme hydrolysis was carried out using the DNS method. The protein estimation of the supernatant after immobilisation was performed using the Bradford method [20].

The assay for the immobilised enzyme was performed for 30 min at 60°C at pH 4.0 using a reaction mixture containing 2% of substrate (CMC) and 0.5 mL of immobilised enzyme (about 20 CMC units). The concentration of glucose was determined using the DNS method. One unit of enzyme activity is defined as 1 μmol of glucose liberated per minute of enzyme assay. All experiments were conducted in triplicate reported as mean values plus or minus the standard deviation.

#### Characterisation of immobilised enzyme and biomass using attenuated total reflection Fourier transform infrared (ATR-FTIR)spectroscopy and scanning electron microscopy (SEM)

The binding of cellulase onto the magnetic nanoparticle supports was determined using ATR-FTIR spectroscopy. The spectrum was recorded using an FTIR spectrometer (BrukerOptik GmbH, Ettingen, Germany). The detector was deuterated triglycine sulfate (DTGS) with a single-reflection diamond ATR sampling module (Platinum ATR QuickSnap™). The scanning range was from 2,200 to 400 cm^-1^ with a scanning resolution of 4 cm^-1^ and 64 scans per sample, and the results were analysed using the OPUS 6.0 suite (Bruker) software.

The untreated and pretreated hemp hurd biomass (HHB) samples were characterised by TEM using a microscope (Zeiss Supra 55 VP, Oberkochen, Germany). The samples were mounted on an aluminium stub, sputtered with gold and allowed to set under vacuum overnight. The imaging was done at an accelerating voltage of 7 kV using a secondary electron (SE2) detector.

#### Determination of enzyme kinetics

The kinetics study of the free and immobilised enzymes was conducted using different concentrations of CMC substrate (0.5% to 2.5%, w/v). The enzyme assays for the free and immobilised enzymes were performed at 50°C and 60°C, respectively, using 0.1 M sodium acetate buffer at pH 4.0. The data analysis was performed with GraphPad Prism 6 software using a Michaelis-Menten kinetic derivation.

#### Thermostability and storage study

The thermal stability of the free and immobilised enzymes was determined at a selected temperature (80°C) in the absence of substrate. The enzyme assays for the immobilised and free enzymes were performed at intervals of 2 h and 30 min, respectively. The immobilised enzyme was stored at 4°C and its activity measured after an interval of 1, 5, 7 and 45 days. The activity was measured via the CMC assay.

#### Reusability of immobilised enzyme

The reusability of the immobilised enzyme was determined by enzyme assay at 60°C. The immobilised preparation was washed with deionised water followed by enzyme assay buffer. After each cycle of the assay was performed, the immobilised nanoparticles were resuspended in buffer and CMC substrate solution. The activity obtained in the first cycle for the immobilised enzyme was taken as the control and represents 100% activity.

#### Hemp hurd pretreatment

The HHB used for the study was obtained as an industrial residue from Commins Stainless Manufacturing (Whitton, NSW, Australia). The hemp hurds were pretreated at high temperature and pressure to remove lignin, ash and other residual components, and also to open the hurd structure to improve enzyme accessibility. Pretreatment was conducted as per the optimised study [10]. The HHB was milled to 1 mm using a cutting mill and then dried at 70°C to obtain a constant weight. The pretreatment slurry was prepared by adding milled HHB at a solid loading of 1%, w/v in sodium hydroxide solution and then autoclaved (121°C, 20 min). The pretreated HHB was washed five times to remove alkaline traces and stored at 4°C after attaining constant weight after drying.

#### Enzyme saccharification of biomass

Enzyme saccharification of pretreated biomass and CMC was performed with the free and immobilised enzymes. Hydrolysis was carried out for 48 h using 0.1 M sodium acetate buffer and a substrate blank at pH 4.0 for both cases. The optimised temperatures for carrying out enzyme hydrolysis for the free and immobilised enzymes were found to be 50°C and 60°C, respectively. The samples were removed at 12-h intervals and tested for reducing sugars. The hydrolysis percentage of cellulose was calculated using the following formula [21]:

$$\begin{array}{ll} \;\mathrm{Cellulose}\;\mathrm{digested}\left(g\right)= & \mathrm{glu}\cos e\;\mathrm{concentration}\\ & \times v\;\left(\mathrm{total}\;\mathrm{reaction}\;\mathrm{volume}\right)\\ & \times 0.9\left(\mathrm{correction}\;\mathrm{factor}\right) \end{array}$$

$$\begin{array}{ll} \;\mathrm{Cellulose}\;\mathrm{hydrolysis}\;\left(\%\right)= & \frac{\mathrm{Amount}\;\mathrm{of}\;\mathrm{cellulose}\;\mathrm{digested}}{\mathrm{Amount}\;\mathrm{of}\;\mathrm{cellulose}\;\mathrm{added}}\\ & \times 100 \end{array}$$

## Results and discussion

**Figure 1:**
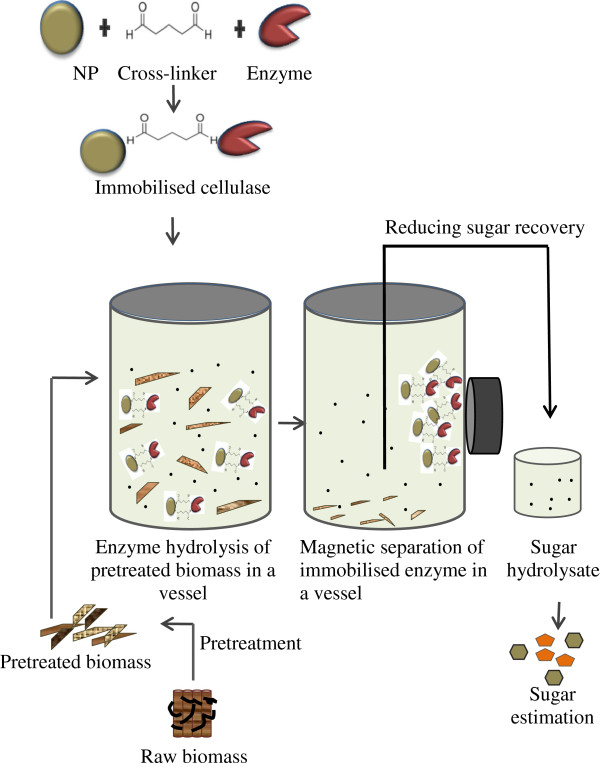
Use of nanoparticle (NP) immobilized enzyme in hydrolyzing HHB for biofuel production.

The hemp hurds used in the present study were composed of 77% holocellulose, 8 to 10% total solids and 13% moisture. The pretreatment of hemp hurds enabled opening of the HHB structure by removing lignin and residual components. The pretreatment resulted in superior hydrolysis of the biomass during enzyme saccharification to produce reducing sugars [10]. The current study focusses on the utilisation of nanoparticle immobilised cellulase for the hydrolysis of natural substrates for the production of sugars. The proposed application will help in the creation of a biorefinery offsetting the biofuel production cost. A detailed flow of the process is provided in Figure 1.

### Characterisation of magnetic nanoparticles

X-ray diffraction (XRD) results showed that the magnetic nanoparticles consisted of a mixture of hematite (Fe_2_O_3_,) and ferrite (Zn_0.4_Fe_2.6_O_4_) [22]. Hematite is a very weak magnetic material. Nevertheless, the saturation magnetisation value was 109 emu/g at 50 kOe, which is considerably higher than the value for undoped Fe_3_O_4_ (typically <60 emu/g) despite the mixed crystal phases. The magnetic hysteresis loop of the nanoparticles showed that the coercivity is zero, indicative of their superparamagnetic nature. This ensured a stable dispersion of nanoparticles in the absence of an external magnetic field so as to sustain a high surface area in which efficient enzymatic reactions could occur. TEM studies indicated that the nanoparticles were near-spherical in shape with diameters of about 40 nm. After immobilisation the size of the nanoparticles became larger, and the preparation was found to be in the form of a dense agglomerate under TEM. This observation supports our FTIR studies of successful immobilisation indicating that the enzyme was able to bind on the activated nanoparticle.

### Binding efficiency of immobilisation

**Figure 2:**
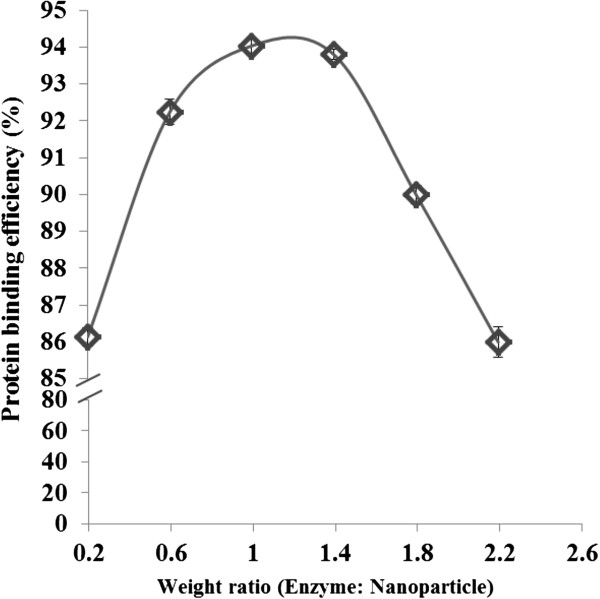
Binding efficiency of cellulase onto nanoparticle with varying concentration of protein.

The binding efficiency and protein loading of cellulase onto the magnetic nanoparticles were confirmed using the Bio-Rad protein assay kit. The immobilisation was done for different protein:nanoparticle ratios, as shown in Figure 2. The activation of nanoparticle supports was tested for 1 h as optimised earlier [18]. The quantity of protein loading and the binding time were studied over 3.5 h (data not shown). The binding rate of protein onto the nanoparticle supports increased for 1.5 to 2 h, and thereafter protein elution slowed, indicating the onset of equilibrium. Therefore, 2 h was the optimum time for protein:nanoparticle immobilisation at 25°C. The experiment was performed in a broad range of protein:nanoparticle concentrations; however, only the best result of the study is presented. Moreover, the minimal binding of 86% was observed with a protein:nanoparticle ratio of 0.2. The binding of protein onto nanoparticles showed a broad range of binding efficiencies, varying in the protein:nanoparticle ratio between 1 to 1.8, and indicating that the activated nanoparticle had attained protein loading saturation. The protein elution concentration was observed to be comparatively high when the protein:nanoparticle ratio increased to 2.2, indicating that the protein concentration was high to bind on the surface of the nanoparticle.

The maximum percentage of binding was obtained at a protein:nanoparticle weight ratio of 1:1, at which 94% binding was achieved. Previous research conducted on immobilising cellulase on a magnetic nanomaterial found 95% binding after a long incubation of 7 h [23]. The efficiency obtained after immobilisation was found to be 86.3%. The immobilisation studies conducted on another nanomaterial found the optimal cellulase immobilisation time to be 30 min on a 7.5 min activated support at a temperature of 40°C [24]. In the present study we detected protein elution after 30 min of immobilisation that gradually lowered with time. Statistically significant elution was not observed after 2 h of incubation. These observations on protein binding were superior to those observed by studies conducted using magnetic nanoparticle supports [25].

### Characterisation studies

**Figure 3:**
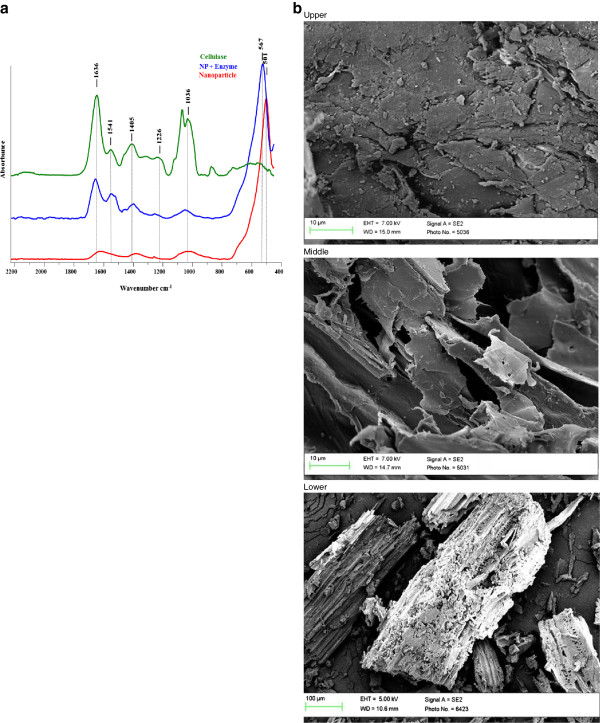
(a) FTIR spectra confirming enzyme immobilization, and (b) SEM images of untreated (upper), alkaline pretreated (middle) and enzyme treated (lower) hemp hurd.

The binding of cellulase onto activated nanoparticles was confirmed by FTIR spectroscopy analysis. The FTIR spectra in Figure 3a represent the spectra of activated nanoparticles, cellulase-bound nanoparticles and of cellulase. A stretch in the peak around 1636 cm^-1^ and 1036 cm^-1^ and modification of the peak from 1541 cm^-1^ to 1226 cm^-1^ on cellulase-bound nanoparticles resemble the peaks in the cellulase enzyme. This characteristic shift in the frequency of cellulase-bound nanoparticles from the activated nanoparticles is suggestive of covalent binding of cellulase onto the nanoparticle. The frequency in the vicinity of 1636 cm^-1^ and 1541 cm^-1^ suggests the stretching of C = O and C-O groups. The stretching pattern near 1541 cm^-1^ also suggests binding of the carboxyl group in the enzyme and amine group of the nanoparticles [25]. The immobilisation of cellulase onto glutaraldehyde activated nanomaterial support resulted in a maximum of 94% enzyme binding efficiency in 2 h of incubation at 25°C.The SEM images of untreated, alkaline pretreated and enzyme hydrolysed hemp hurd are shown in Figure 3b. The structure of the untreated biomass is compact and rigid, although after pretreatment this structure becomes fractured and exposed. The structure opens into long, rod-shaped transverse vessels which demonstrate the exposure of tracheids in the HHB and make the structure easily accessible to enzymes. After enzyme hydrolysis, it was observed that the biomass structure was fractured into small pieces, indicating disruption of the rigid structure and erosion of the prominent features obtained after pretreatment.

### Enzyme activity at varying pH and temperature

**Figure 4:**
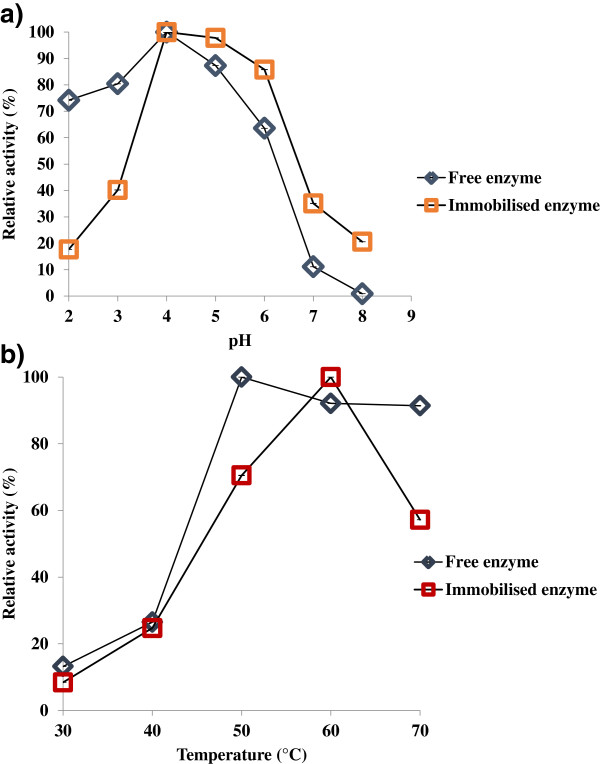
(a) Effect of pH and (b) temperature on the activity of free and immobilised enzyme.

The pH profile demonstrating the relationship between pH buffer and catalytic activity of free and immobilised enzymes is represented in Figure 4a. The enzyme activity of the free and immobilised enzymes was observed to peak at pH 4.0 in sodium acetate buffer (0.1 M). The activity of both types was found to gradually reduce with increasing pH. The immobilised enzyme was found to be more stable in the pH range of 4.0 to 5.0, within which insignificant differences in activity were observed. A similar pH stability observation has been reported while conducting immobilisation of cellulase onto an acrylonitrile copolymer nanofibre, where the immobilised cellulase activity was found to increase from pH 3.0, attain stability between pH 4.0 to 5.0, and subsequently decrease again with higher pH values [26]. Another study conducted on immobilised cellulase demonstrated that the enzyme preparation was quite stable in a wide pH range (pH 1.5 to 12.0) but the activity of natural/free cellulase was found to be reduced for pH 6.0 [27]. The studies conducted on cellulase immobilisation on cation-exchange membranes demonstrated a broader pH range of 3.8 similar to the present work [28].

The enzyme activity of the free and immobilised enzymes was investigated at various temperatures ranging from 30 to 60°C, in an attempt to understand the effects of temperature on the activity of cellulase following immobilisation. Figure 4b demonstrates the increase in enzyme activity with temperature in both the free and immobilised forms. The relative enzyme activity increased up to a temperature of 50°C for the free enzyme while it increased up to 60°C for the immobilised enzyme, thereafter declining with further increases in temperature. The temperature profile study demonstrated that the optimum temperatures for the free and immobilised enzymes were 60°C and 50°C, respectively. For the immobilised enzyme, about 60% enzyme activity was retained in the 50 to 70°C range, with the activity peaking at 60°C. Immobilisation increased the resilience of the enzyme and improved the thermal stability at higher temperatures, a finding which echoes the results of a previous study where cellulase enzymes attained stability at 50°C [24]. An earlier study conducted on immobilisation using cellulase complex binding onto magnetic nanoparticle supports showed optimum activity at 50°C [25]. The present study demonstrated that the stability of the enzyme increased by 10°C with immobilisation, indicating improved thermal stability.

### Kinetic study on immobilised and free enzymes

The kinetic study of the free and immobilised enzymes was done using varying concentrations of CMC. The rate of reaction was monitored with respect to substrate concentration using a Michaelis-Menten kinetic derivation based on a non-linear regression. It is considered to be one of the best models to study enzyme kinetics at varying substrate concentration where rate of reaction is plotted against concentration. The *K*_*M*_ (half-maximal velocity) and *V*_*max*_ (maximal velocity) values obtained using GraphPad Prism 6 software (the software has a built-in feature to determine enzyme kinetics) at 95% confidence is given in Table 1. The K_*M*_ values of the free and immobilised enzymes are 0.87 and 2.6, respectively. A threefold increase in the K_*M*_ value of immobilised versus free enzyme was observed. Similar results were observed in a previous study, where the K_*M*_ values increased about 2.7 times following immobilisation of cellulase onto a nanomaterial support [29]. Other reports on immobilising cellulase on various supports have suggested that the K_*M*_ value of the enzyme changes as a result of immobilisation [30].

**Table 1:** Rate of reaction of free and immobilised enzyme at different substrate concentrations using Michaelis-Menten kinetics

	**Free enzyme**	**Immobilised enzyme**
***V***_***max ***_ ***(mg/mL/min)***	0.72 ± 0.1	2.0 ± 0.6
***K***_***m ***_ ***(mg/mL)***	0.87 ± 0.3	2.6 ± 1.3

95% confidence level (GraphPad Prism 6).

Increases in the K_*M*_ value following BGL immobilisations on gamma-Fe_2_O_3_@SiO_2_ core-shell magnetic nanoparticles have also been reported [31]. The minor change in apparent *K*_*M*_ values suggested that the substrate binding affinity of the enzyme active site was altered by immobilisation. The *K*_*M*_ values for the free and immobilised enzyme BGL *Agaricus arvensis* were 2.5 mM and 3.8 mM, respectively [32]. This experiment provided preliminary data on cellulase efficiency variation with substrate concentration and the impact of immobilisation, and aided in the design of enzyme hydrolysis experiments with natural and synthetic substrates.

A similar study conducted on cellulase immobilisation using the Michaelis-Menten kinetic model reported rate constants *K*_*M*_ and *V*_*max*_ using synthetic microcrystalline cellulose [33].

### Thermal stability of the free and immobilised enzyme

**Figure 5:**
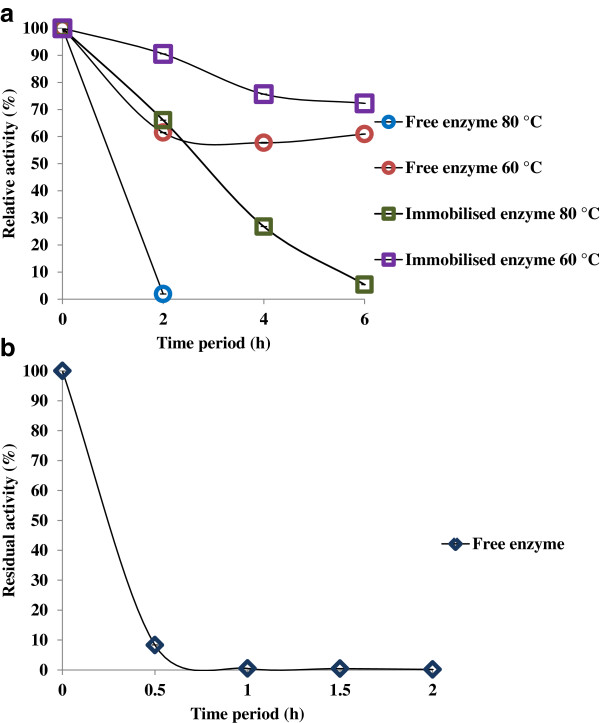
(a) Thermostability study of free and immobilised enzymes and (b) free enzyme at 80°C.

An improvement in the stability of the immobilised cellulase at higher temperatures is shown in Figure 5a. The results obtained from the thermal stability studies show that the immobilised enzyme was stable for approximately 4 h at 80°C. It retained about 66% of its initial activity in the first 2 h of incubation, but this gradually decreased in the next 4 h of incubation, providing a maximum of 26% activity after 4 h. After an incubation of 4 h, the enzyme had lost about 73% of its activity. The immobilised preparation completely lost activity by 6 h of incubation at 80°C. The loss of activity for the immobilised cellulase preparation was slower than that for the free enzyme. The activity of free cellulase was found to decrease in the first half hour of incubation, which suggests that the free enzyme denatured at 80°C, as shown in Figure 5b. The loss in activity of the free cellulase over 2 h of incubation at 80°C is shown in Figure 5b. The immobilised enzyme retained 72% of its initial activity for up to 6 h of incubation at 60°C. The activity was 90% after the first 2 h of incubation and gradually reduced to 75% after 4 h of incubation, thereafter reducing to a negligible rate up to 6 h. The enzyme was more stable at 60°C after immobilisation than it was at 80°C. The free enzyme activity reduced to 61% after 2 h of incubation, and thereafter no significant decrease in the activity from 4 to 6 h was observed. This indicated that the free enzyme retained about 60% of its initial activity for up to 6 h of incubation at 60°C. These results indicate that the properties of the enzyme were not impacted by immobilisation, with the exception of higher stability at elevated temperatures. A similar study demonstrated the stability of the immobilised preparation at 80°C for an hour; however, the natural cellulase lost its activity during the same time period, similar to our observation [27]. In previous reports it has been seen that the stability of the enzyme preparation on magnetic nanosized supports has reduced to about 50% after an incubation of 2 h [23].

In addition to better thermal stability, immobilisation provides storage stability by binding the enzyme onto a support and inhibiting denaturation over time. The immobilised cellulase preparation was stored at 4°C in acetate buffer (0.1 M, pH 4.0) for a period of seven weeks and the activity was measured at intervals of 1, 5, 7 and 45 days (data not shown). The immobilised enzyme retained almost full activity over the entire period, thus demonstrating that the immobilised enzyme preparation retained activity without significant loss during the storage.

### Reusability of immobilised enzyme

**Figure 6:**
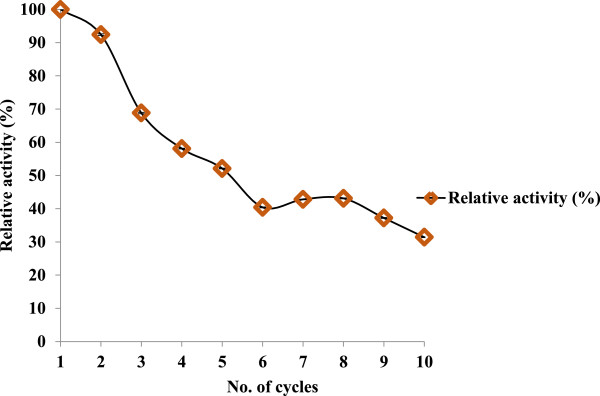
Reusability study of immobilised enzyme using CMC substrate.

Immobilisation of cellulase can facilitate enzyme recycling in a sequential batch-wise process, thereby lowering the enzyme cost. The immobilised enzyme was stable for up to seven consecutive cycles at 60°C of CMC hydrolysis for 30 min; thereafter, the activity reduced significantly (Figure 6). The immobilised enzymes maintained about 70% of their activity until the third cycle. Earlier studies of cellulase immobilisation on polyamidoamine (PAMAM)-grafted silica reported that 75% activity was retained after three cycles. In another study, 41% activity was retained after six cycles when the enzyme was attached using adsorption and 67% when the enzyme was covalently cross-linked [34]. Some reports have suggested that the gradual loss of enzyme activity after only a few cycles occurs due to factors such as product inhibition, structural modification of the enzyme, protein denaturation and/or inactivation of the enzyme [25]. Since the nanoparticle was magnetic in nature, it facilitated easy separation and recovery of the immobilised enzyme from the reaction mixture, thus supporting reusability.

### Hydrolysis of CMC using free and immobilised enzymes

**Figure 7:**
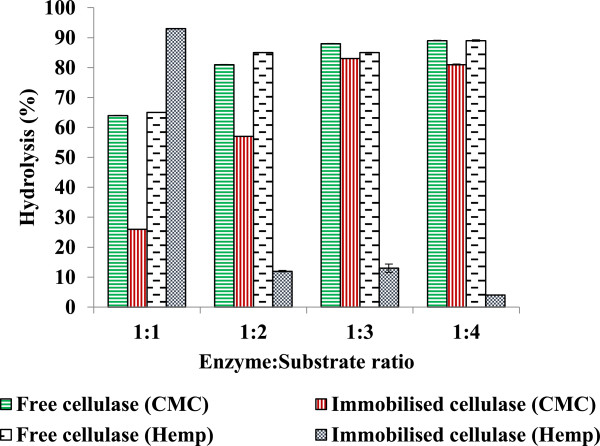
Hydrolysis of CMC and pretreated hemp hurd using free and immobilised enzyme for 48 h.

Free and immobilised cellulase were used up to 48 h to hydrolyse varying concentrations of untreated synthetic substrate (CMC), ranging in enzyme:substrate ratio from 1:1 to 1:4, as shown in Figure 7. Hydrolysis increased with enzyme:substrate ratios from 1:1 to 1:3 for both free and immobilised enzymes, with no significant increase observed at a 1:4 ratio of enzyme:substrate. The immobilised and free enzymes showed optima at a 1:3 enzyme:substrate ratio, with the immobilised enzyme hydrolysing 83% of substrate and the free enzyme hydrolysing 88% of substrate over 48 h. The level of hydrolysis at a 1:1 ratio was 26% for the immobilised enzyme, increasing to 57% with higher levels of CMC. With the free enzyme at the same 1:1 enzyme to substrate ratio, the level of hydrolysis was 17%. When the level of CMC was increased to a 2:1 ratio of substrate to enzyme, hydrolysis increased to 81%. During a previous study, researchers obtained 85% glucose yields using free enzymes, and a maximum of 83% glucose yield with immobilised cellulase when hydrolysing CMC pretreated with ionic liquid [35]. An earlier study hydrolysing CMC resulted in a lower yield with immobilised compared with free enzyme and concluded that dilution of enzyme on the support accounted for the lower yield after immobilisation [36]. Another study conducted using immobilised cellulase to hydrolyse ionic liquid treated cellulose found that the addition of 1-ethyl-3-methylimidazolium diethyl phosphate (EMIM-DEP) increased the hydrolysis rate by a factor of 2.7, resulting in 0.95 g glucose/g cellulose in 8 h of hydrolysis with the addition of 4% of EMIM-DEP [37].

### Enzymatic saccharification of hemp hurd biomass (HHB)

Varying concentrations of pretreated HHB (0.5 to 4 mg) were incubated with free and immobilised enzymes (5 mg protein). Enzyme saccharification at 48 h resulted in a maximum of 89% hydrolysis using free cellulase, however, 93% hydrolysis was achieved with immobilised cellulase at a 2:1 enzyme:substrate ratio. Enzyme saccharification using the immobilised enzyme was found to reduce with an increasing biomass concentration. Hydrolysis reduced when the enzyme:substrate ratio was increased from 1:1 to 1:2 (Figure 7). Partial hydrolysis resulted either from reduced accessibility of cellulase with increasing substrate concentration, or from interference of lignin during the hydrolysis. With increased shaking, the enzyme biomass interaction did not improve. The rate of hydrolysis initially increased over a 24-h period, thereafter stabilising for both the free and immobilised enzymes. The free enzyme exhibited an optimum hydrolysis of 85% at a 1:3 enzyme:substrate ratio. Similar observations were reported for a study on enzyme saccharification of ionic liquid pretreated yellow poplar using immobilised enzyme. This study gave a maximum of 45.3% hydrolysis in 24 h, with no improvement observed even after a second addition of cellulase preparation [38]. A study with immobilised enzymes using glutaraldehyde as a cross-linker, applied to the hydrolysis of steam-exploded corn stalk and bagasse substrate, demonstrated that 24 h of hydrolysis was optimum and that a longer hydrolysis time did not improve the yield [39]. The present study exhibited better results compared to previous studies [36,37], which were conducted at 60°C and pH 4.5. Although the reaction conditions of the present study were similar to those of earlier studies, lower enzyme:substrate ratios for enzyme saccharification were optimised. A study employing cross-linked glutaraldehyde *Aspergillus niger* cellulase demonstrated that after immobilisation the activity increased by 15% and provided 52% of enzyme saccharification of rice hull [40]. Recently, another study demonstrated continuous hydrolysis of waste bamboo when cellulase was immobilised on silica through the assistance of L-cysteine functionalised gold nanoparticles [41].

## Conclusions

The immobilisation of cellulase onto a functionalised nanoparticle was achieved and used to investigate the hydrolysis of a synthetic (CMC) and a natural pretreated substrate (HHB). The confirmation of cellulase and nanoparticle binding (maximum 94%) was done using FTIR spectroscopy. The comparative assessment of the effects of pH and temperature on free and immobilised enzymes demonstrated superior stability for the immobilised enzyme at elevated temperature. The thermostability of the immobilised enzyme increased to 80°C, and it retained 50% of its initial activity for up to five runs with superior storage stability (45 days). An optimum of 88% CMC hydrolysis and a maximum of 89% hydrolysis with pretreated HHB was obtained using the free enzyme. The immobilised enzyme provided successful hydrolysis of 83% with CMC and 93% with hemp hurd biomass. There is an opportunity to further improve the hydrolysis percentage of biomass during enzyme saccharification using immobilised enzymes at higher substrate ratios.

## Abbreviations

CMC: carboxymethyl cellulose; FTIR: Fourier transform infrared spectroscopy; HHB: hemp hurd biomass; SEM: scanning electron microscopy.

## Competing interests

The authors declare that they have no competing interests.

## Authors’ contributions

REA carried out the research work and drafted the manuscript. REA and MV participated in the design and performance. CB helped to polish the manuscript. MP conceived the study and participated in its design and coordination, and helped to draft the manuscript. All authors read and approved the final manuscript.
